# Street Noises

**Published:** 1895-11-30

**Authors:** 


### Editor's Letter-Box.

[Our correspondents are reminded that prolixity is a great bar to publication, and that brevity of style and conciseness of statement greatly facilitate early insertion.]

### STREET NOISES.

The Secretary of the Association for the Suppression of Street Noises writes: The action of the Vestry of Kensington to which you allude is a very commendable one, but the London County Council is hardly likely to recede from the position it took up in 1892, when, acknowledging that it had the power, it refused to pass bye-laws for the suppression of street music and other noises on the petty and contemptible ground that it had not the control of the police. But it must be remembered that if it takes a different attitude now, relief will only be afforded to the County of London, and even the City will not be affected. There are many residing just on the fringe, in Croydon, Sutton, Richmond, Twickenham, and other places who need relief. And besides that, persons in Bristol, Birmingham, and other large towns are clamouring for relief. I shall be glad if you would render your valuable assistance to the Association for the Suppression of Street Noises which has recently been established. Our aim is to obtain legislative relief for the whole of England and Wales. A large and influential committee has been formed, and the support of a large number of members of Parliament obtained; a draft Bill to be put before the Legislature has been approved, and a large measure of support has been promised. I shall be much obliged if you will allow me through your columns to invite the co-operation of those who know the direful effects of street music and street noises on the sick and suffering, and if they will kindly give me their experience, and support us in our work, I shall be happy to have their co-operation.

